# The revised Healthy Purchase Index (r-HPI): a validated tool for exploring the nutritional quality of household food purchases

**DOI:** 10.1007/s00394-022-02962-4

**Published:** 2022-08-27

**Authors:** Marlène Perignon, Pascaline Rollet, Marion Tharrey, Daisy Recchia, Sophie Drogué, France Caillavet, Caroline Méjean, Nicole Darmon

**Affiliations:** 1 MoISA, Univ Montpellier, CIHEAM-IAMM, CIRAD, INRAE, Institut Agro, IRD, Montpellier, France; 2 ALISS, INRAE, Univ Paris-Saclay, Ivry, France

**Keywords:** Nutrition, Food supply, Score, Indicator, Validity, Diet quality, Receipts, Food shopping behaviours, France, Grocery purchases

## Abstract

**Background:**

The Healthy Purchase Index (HPI) assesses the nutritional quality of food purchases (FP) from food group expenditure shares only. However, it was developed from the FP of a disadvantaged population.

**Objective:**

To adapt and validate the HPI for a general population.

**Methods:**

FP were obtained from a representative sample of French households (Kantar WorldPanel) subdivided into two subsamples. The first sample (*n* = 4375) was used to adapt and validate the score; the second sample (*n* = 2188) was used to test external validity. The revised-HPI (r-HPI) includes 2 subscores: the diversity subscore and the quality subscore. Diversity subscore points were awarded when expenditure shares were above the 25th percentile for 5 food groups (“Fruits”, “Vegetables”, “Starches”, “Dairy”, “Meat, Fish and Eggs”). Regression models between the expenditure shares of each food group and the Mean Adequacy Ratio (MAR) and the Mean Excess Ratio (MER) of FP were used to select quality subscore components and define cut-offs for point allocation. Construct validity was assessed on the first sample using Spearman’s correlations between the r-HPI and the four nutritional quality indicators (NRF9.3, MAR, MER, energy density), and also by comparing the r-HPI of monthly FP of sub-populations defined by criteria known to influence diet quality (age, gender, income, education) and between households having a monthly food basket of higher (MAR > median and MER and energy density < median) vs. lower nutritional quality within the population, using Wilcoxon tests or pairwise comparisons of contrasts. External validity was tested by performing the same analyses on the 2nd sample of 2188 households.

**Results:**

The adaptation led to include new components (e.g. red meat) and define new cut-offs (e.g. − 1 point when budget share for red meat > 21%). The r-HPI (mean = 6.50 ± 3.58) was strongly correlated with NRF9.3, MAR, MER and energy density (0.59, 0.52, − 0.41 and − 0.65, respectively, *p* < 0.01) and poorly correlated with total energy content (− 0.096, *p* < 0.001). The r-HPI was significantly higher in women (*β* = 1.41 [0.20], *p* < 0.01), households having a food basket of higher nutritional quality (*β* = 4.15 [0.11], *p* < 0.001), and increased significantly with age, income and education levels. Similar results were obtained in the 2nd sample.

**Conclusion:**

We showed the validity of the r-HPI in a large sample of French households. As it does not require food quantity or nutrient content, it can be used as a valuable tool to explore FP behaviours. Cut-offs can be used in health promotion to provide nutri-economic counselling.

**Supplementary Information:**

The online version contains supplementary material available at 10.1007/s00394-022-02962-4.

## Introduction

Food behaviours can be influenced not only by individual factors (e.g. age, gender or socio-economic characteristics) but also by environmental factors such as food availability and accessibility to food outlets. Accordingly, nutrition interventions targeting food purchasing behaviour are increasingly used to promote healthier diets [1, 2]. In this context, it is crucial to better understand the role of the food environment, defined as the physical, organizational and sociocultural space in which people encounter meals and food [3], in promoting healthy food behaviours. Being at the interface between the food environment and eating behaviours, food purchases (FP) can help research these relationships [4].

FP data offer various advantages for studying dietary behaviours. Unlike food consumption surveys based on 24 h recalls or food frequency questionnaires, FP data are an objective measure of household behaviour, free of memory bias. They depict the home food environment that directly influences the quality of diet of household members. Appelhans et al*.* showed that objectively documented household FP provide an unbiased and reasonably accurate estimate of overall diet quality [5]. Moreover, receipts can provide in-depth information about purchased food items such as price, production mode (conventional vs. organic) or food stores types and locations, thus allowing an analysis of food costs or food purchasing behaviours (frequency of visits to food outlets, food trips, etc.).

Different studies, mainly based on mean food prices, have shown the contradiction between nutritional adequacy and affordability of diets, healthier diets being associated with higher costs, and cheaper and more energy-dense diets being chosen by lower income groups [6]. Consequently, FP data are of great interest when exploring the relationship between nutritional quality and diet cost based on real food expenditures, rather than estimated based on mean food prices. Moreover, as higher nutritional quality diet can be obtained at no additional cost with specific food choices [7], FP data can be used to develop tools to guide consumers with economic constraints towards healthier choices when purchasing food [8, 9].

Yet, despite the great potential of FP data and the key role of nutrition in sustainability, the nutritional quality assessment of FP remains limited [10]. This could be partly explained by the laborious work entailed in collecting FP data and the pairing of purchased food items with food composition databases. Relying on food composition databases also requires converting the quantity of purchased food items into quantity “as consumed”, thus adding an additional barrier since conversion factors are not readily available. Moreover, missing information on quantities on receipts could hinder the use of FP data to estimate the nutritional content of food baskets.

In this context, we previously developed the Healthy Purchase Index (HPI), a scoring system based on FP from a convenience sample of low-income French households, to directly assess the nutritional quality of FP using expenditure shares of food groups in total food expenditures rather than food quantities [11]. This score overcomes the barriers of requiring information on quantity or nutritional content of food items. However, the previous HPI was developed from the purchasing data of approximately 100 households from a disadvantaged population that may have specific food purchasing patterns, and its adaptation and validity in a large general population with more diverse purchasing behaviours remained to be evaluated. In addition, because the environmental impact of diet is of great concern, the updated French dietary guidelines now integrate sustainability considerations [8], and include recommendations on food groups that were not included in the previous HPI, such as recommendations on red and processed meats.

The objective of the present study was to adapt and validate the previously developed HPI for the general population based on a large survey of French households’ FP, and to update it according to the new French dietary guidelines.

## Methods

### Sampling and food purchase data

Sociodemographic and FP data were obtained from a representative consumer panel of 6565 French households, the Kantar Worldpanel 2014 [12], who reported all of their purchases (expenditure and quantity purchased by food item) made during the week or on weekends for home consumption, from various distribution channels (e.g. supermarkets, markets, producers, etc.) for at least 25 weeks over a year. This panel does not take into account purchases made in restaurants, coffee shops and bakeries. Households who did not report 4 consecutive weeks of purchases were excluded (*n* = 2). The resulting sample of 6563 households was then divided into two randomly selected subsets of households to make up a first sample dedicated to the adaptation and internal validation of the score (*n* = 4375), and a second sample used for external validation (*n* = 2188). In the present study the two samples are, respectively, referred to as “adaptation sample” and “external validation sample”. The HPI was previously developed based on monthly FP. This time period is a compromise between representativeness of FP, in particular considering the frequency of salary payments, and feasibility of data collection for participants. For each household, 4 consecutive weeks were then randomly selected among their annual purchases to constitute a monthly food basket.

The following socio-demographic data were used to characterize the adaptation and validation sample: age, education level (categorized in 4 classes: primary and secondary school, high school, higher secondary school, Bachelor or Master degrees or higher) and socio-professional category (8 classes) of the respondent, number of household members, income per consumption unit, household structure (categorized in 4 classes: 1 adult, 1 adult with child[ren], several adults, several adults with child[ren]), and residence area (8 regions).

### Nutritional quality assessment

#### Matching the food products purchased to the food composition database

Each food product purchased by the Kantar Worldpanel was linked to the closest food item from the extended version (CALNUT) of the French food composition database CIQUAL 2016 [13]. For example, all food products classified in the Kantar database as “mashed potatoes” with the descriptors “with milk” or “with cream” were associated to the CIQUAL food item “Potatoes, dehydrated flakes, with milk or cream”. Food items (*n* = 1328) were classified into 11 groups and 27 subgroups (detailed in Supplemental Table S1). In particular, attention has been paid to distinguish “Red meat” from “Processed meat” subgroups to take into account the new French dietary guidelines that now include recommendations for these subgroups. As updated dietary guidelines also include a specific recommendation for legumes, it was initially planned to distinguish this subgroup. However, because their expenditure share was so low in the studied population (mean = 0.24%, median = 0.00%), it was eventually decided to include legumes in the “Unrefined Starches” subgroup. Considering that condiments (salt, pepper, spices, etc.) are generally bought for consumption over several months and that their contribution to energy intake is low, the “Condiments” group was not included in the analyses. As in the previous HPI, “Water” and “Baby food” groups were also excluded from analyses, as these groups represented a small share of total food expenditure (each < 1.5%) and were purchased by a small proportion of the population (90% of households had expenditure < 4%).

#### Energy and nutrient contents of food baskets

Quantity purchased of each food item was converted into quantity “as consumed” using a conversion factor that accounts for weight change due to peeling, bones, cooking, etc. Conversion factors were previously estimated by calculating the ratio of net quantities to raw quantities provided for each food item in the Second French Individual and National Study on Food Consumption (INCA2) [14] followed by a verification of each ratio by a dietician.

Total energy and nutrient contents of the monthly food basket were estimated for each household by multiplying the quantity “as consumed” of each food item purchased by that household by its nutrient contents (macronutrients, vitamins, minerals, fatty acids, fibres) derived from the CALNUT database, and summing over all energy and nutrient contents over the 4-week period.

#### Nutritional quality of food baskets

The overall nutritional quality of food baskets was assessed using 4 indicators: the Nutrient-Rich Foods Index 9.3 (NRF9.3), an index based on 9 nutrients to encourage and 3 nutrients to limit [15], the Mean Adequacy Ratio (MAR), an indicator of overall good nutritional quality [16], the Mean Excess Ratio (MER) which assesses the excess intake of 3 nutrients to limit, and the Solid Energy Density (SED), known to be related to poor nutritional quality [17, 18].

The NRF9.3 was calculated for 100 kcal as described by Fulgoni et al*.* [15] as the sum of the daily values of nutrients to encourage and subtract the daily values for nutrients to limit.

The MAR was calculated for 2000 kcal of purchases for each household as the mean percentage of daily recommended intakes [19] for 23 key nutrients (proteins, fibre, linoleic acid, linolenic acid, DHA, vitamin A, thiamin, riboflavin, niacin, vitamin B6, folates, vitamin B12, ascorbic acid, vitamin E, vitamin D, calcium, potassium, iron, magnesium, zinc, copper, iodine, and selenium) as previously described [18].

The MER was calculated for 2000 kcal of purchases for each household as the mean percentage of the maximum recommended values (MRV) for three nutrients to limit, namely saturated fatty acids (SFA), sodium and free sugars, as previously described [20]. The MRV for SFA and free sugars corresponded to 12% [21] and 10% [22] of a standard energy intake of 2000 kcal, i.e. 26.7 g and 50 g, respectively [19]. In the absence of MRV for sodium, the median sodium intake in the French population (2633.5 mg/d, based on data from the second Individual and National Study on Food Consumption) was used as the MRV, as recommended by the French Agency for Food, Environmental and Occupational Health & Safety (ANSES) [21]. Unlike the previously published MER, each nutrient excess ratio lower than 100 was not truncated to 100 to avoid non-normal distribution of the indicator.

The SED (in kcal/100 g) was calculated by dividing the total energy provided by solid foods by their total edible weight. As suggested by Ledikwe et al. [23], foods typically consumed as beverages (e.g. milk, juices, and soft drinks) were not included in the calculation.

### Adaptation of the HPI

The food expenditure shares were calculated for each household as the percentage of expenditure for each food group and subgroup in total monthly food expenditures. The food expenditure shares estimated in the first sample of 4375 households were used to adapt the 2 HPI subscores (i.e. the diversity subscore and the nutritional quality subscore) to the general population. Food group and subgroup expenditure shares with non-normal distribution were categorized by 2 classes (purchased/not-purchased or based on the median expenditure share) or in quartiles as appropriate.

#### r-HPI diversity subscore

In the previous version of the HPI, 1 point was allocated when the expenditure share was greater than 5% for 5 food groups: fruits, vegetables, starches, dairy products and meat, fish and eggs (MFE). To better account for the difference in food expenditure shares between the food groups, the single 5% expenditure cut-off has been revised according to the distribution of food expenditure shares for each of the 5 food groups. The 10th and 25th percentiles of expenditure shares have been tested.

#### r-HPI nutritional quality subscore

##### Selection of subscore components

Following the methodology used for the previous HPI version, the first step was to select the food groups and subgroups to include in the nutritional quality subscore based on their association with nutritional quality indicators. First, we performed univariate linear regressions between the expenditure share of each food group and subgroup and the MAR and MER. Only food groups and subgroups associated with the MAR and the MER at 0.20 significance level were retained for inclusion in the multivariate linear regression models. Food groups and subgroups whose association with both the MAR and the MER were statistically significant at *p* < 0.05 were pre-selected as components of the subscore.

##### Definition of cut-offs for point allocation

The second step consisted of defining the cut-offs used for allocating points. In this revised version of the HPI, the cut-offs were defined for each subscore component based on the breakpoints identified by segmented regression models between the expenditure share of each component and both the MAR and the MER. When a segmented regression model identified a steep regression slope for a segment of expenditure shares that included a high number of households, then deciles of expenditure shares were used to identify intermediate cut-offs.

In addition, multivariate regression models between the 6 food groups corresponding to the subscore components (Fruits and Vegetables; MFE; Dairy; Starches; Fats; Discretionary foods) and the MAR and the MER were performed to identify the groups that contributed the most to nutritional quality, and to balance accordingly the maximal number of points that groups could earn when summing maximal points of their respective subgroups.

### Validity assessment of the revised-HPI (r-HPI)

Content and construct validity of the r-HPI were assessed. To assess content validity [24, 25], we evaluated the correlations between score components and the r-HPI applied to the monthly FP of the first sample of 4375 households from the French Kantar WorldPanel (adaptation sample).

Different attributes (concurrent validity and discriminating capacity) of the construct validity, i.e. the extent to which an index assesses a construct of concern and is associated with evidence that measures other constructs in that domain [25, 26], were evaluated. The concurrent validity of the score was assessed using Spearman correlations between the r-HPI of monthly FP and the 4 indicators of nutritional quality (NRF9.3, MAR, MER, SED), as well as between the r-HPI and the excess ratios of each nutrient included in the MER (SFA, free sugars, sodium). Correlations were also tested against the previous version of the HPI [11]. The discriminating capacity was assessed by comparing the r-HPI of monthly FP of sub-populations defined by criteria known for their association with diet quality, namely age, gender, income, and level of education, by Wilcoxon tests (for age and gender) or pairwise comparisons of contrasts, with Bonferroni adjustment, when comparison was adjusted for age (for income and education levels). For gender, estimates and tests were performed on a subsample of single adult households. Discriminating capacity was also tested by comparing the r-HPI of FP of higher vs. lower nutritional quality within the population. To identify FP of good nutritional quality, we used the positive deviance approach according to which some individuals adopt “positive” (or beneficial) behaviours, although the constraints to which they are submitted and/or the context in which they live should lead them to adopt a “negative” behaviour, like the majority of individuals in the same population [27]. Households were classified as having a food basket of higher nutritional quality when their FP met the following 3 criteria: a higher MAR, a lower MER and a lower SED than the respective median values [20]. The r-HPI of their FP was compared to that of households having a food basket of lower nutritional quality (MAR < median or MER > median or SED > median) by Wilcoxon tests.

We tested the ability to assess the nutritional quality of FP independently of their energy content using Spearman’s correlation between the r-HPI and total energy content of purchases.

The r-HPI external validity was tested by performing the same set of validation analyses (content validity, concurrent validity, discriminating capacity) on the 2nd sample of 2188 households. Differences of socio-economic characteristics between adaptation and external validation samples were tested using Wilcoxon tests (for age of the respondent, number of household members, income per consumption unit, and food expenditure) or Chi-square tests (for household structure, education level, socio-professional category, and residence area). Statistical significance of validity assessment analyses was set at *p* < 0·05.

### Robustness assessment of the r-HPI

Stratified analyses were performed to test the robustness of the r-HPI at different levels of (i) total expenditure, (ii) alcohol expenditure share, (iii) mixed dishes expenditure share (as this food group was not included in the subscore) and (iv) animal to plant protein ratio of the food basket. The associations between the score and nutritional quality indicators (MAR, MER, SED and NRF9.3) were assessed across deciles of these 4 food basket characteristics.

All analyses described in “Methods” were performed with the statistical software R version 3.5.2.

## Results

### Sample characteristics

Sample characteristics are shown in Supplemental Table S2. The mean age of the respondent in the adaptation sample (*n* = 4375 households) was 52.6yrs ± 15.3, and the mean monthly income per consumption unit was 1748€ ± 809€. Households of this sample purchased a total of 455,653 food products and had a mean food expenditure of 299€/four weeks (± 161€).

The socio-economic characteristics (age of the respondent, number of household members, income per consumption unit, household structure, education level, socio-professional category, residence area) and the total food expenditure of the external validation sample (*n* = 2188 households) were not significantly different from that of the adaptation sample (see Supplemental Table S2).

### Adaptation of the HPI to develop the r-HPI

#### r-HPI diversity subscore

The 25th percentile of expenditure shares for the food groups were 2.77% (fruits), 3.50% (vegetables), 2.27% (starches), 8.19% (dairy products) and 19.73% (meat, fish and eggs), respectively. These expenditure shares were used to define cut-off values for point allocation: households with an expenditure share above the cut-off value were attributed 1 point for each of the 5 components, leading to a diversity subscore ranging from 0 to 5 points (Table 1). While 0.6, 2, 10, 22, 38 and 27% of the households obtained a total diversity subscore of 0, 1, 2, 3, 4 and 5 points, respectively, when using the 25th percentile cut-offs, the distribution was 0.0, 0.5, 1.8, 7.8, 27 and 63%, respectively, when using the 10th percentile cut-offs. Therefore, the 25th percentiles of expenditure shares were chosen rather than the 10th percentiles to define point allocation cut-offs since they resulted in a more balanced distribution of households across total subscore levels.

**Table 1:** Components and cut-off values used for the r-HPI computation

Diversity subscore (0–5 points)		
Component	Cutoff value (% of total food expenditure^1^)	Score
Fruits	[0–2.8[	0
	≥ 2.8	1
Vegetables	[0–3.5[	0
	≥ 3.5	1
Starches	[0–2.3[	0
	≥ 2.3	1
Dairy products	[0–8.2[	0
	≥ 8.2	1
Meat/fish/eggs	[0–19.7[	0
	≥ 19.7	1

Quality subscore (− 8 to + 12 points)		
Component	Cutoff value (% of total food expenditure^1^)	Score
Fruits and vegetables	[0–6[	0
	[6–9[	1
	[9–12[	2
	[12–16[	3
	≥ 16	4
Cheese	< 4	1
	[4–8[	0
	≥ 8	− 1
Milk and yogurts	< 2	0.5
	[2–9[	1
	≥ 9	0
Eggs and poultry	< 3	0
	≥ 3	1
Fish	< 1.5	0
	[1.5–4[	1
	[4–7[	1.5
	≥ 7	2
Red meat	≤ 21	0
	> 21	− 1
Processed meat	≤ 6	0
	]6–10[	− 1
	≥ 10	− 2
Fats	Total fats = 0	0
	Total fats > 0 and animal fats [0–1]	1
	Animal fats ]1–2]	0
	Animal fats > 2	− 1
Starches	Total starch = 0	0
	Total starch > 0 and unrefined starch = 0	0
	Unrefined starch ]0–18%[ of total starch	1
	Unrefined starch [18–30%[ of total starch	1.5
	Unrefined starch ≥ 30% of total starch	2
Discretionary foods	< 7	0
	[7–13[	− 1
	[13–18[	− 2
	≥ 18	− 3

^1^Cutoff values are expressed in % of total food expenditures except were specified, e.g. “Unrefined starch ]0–18%[ of total starch”

#### r-HPI nutritional quality subscore

##### Choice of subscore components

The results from the univariate models can be seen in Table 2. The “Alcoholic drinks” group was negatively associated with both the MAR and the MER. This suggested that this group was not relevant as a predictor of nutritional quality since it did not provide nutrients and rather contributed to a “dilution effect”: the higher the expenditure share on alcoholic drinks, the lower the expenditure shares remained for the other food groups. The “Alcoholic drinks” group was therefore not included in multivariate models. All subgroups of the “Fruits and vegetables” and “Discretionary foods” groups were significantly associated with the MAR or the MER and in the same direction (positive or negative) than the association of the corresponding group. Therefore, these two groups were included in the multivariate models, rather than their respective subgroups. For the “MFE”, “Dairy” and “Fats” groups, subgroups were preferentially included over groups because of their nutritional specificities.

**Table 2:** Univariate and multivariate associations between indicators of nutritional quality of food purchases (mean adequacy ratio (MAR) and mean excess ratio (MER) for 2000 kcal) and food group and subgroup expenditure shares (in percentage) for 4375 households from the French Kantar Worldpanel

Groups	Subgroups		Univariate associations				Multivariate associations			
			MAR		MER		MAR		MER	
Fruits and vegetables			0.34	***	− 0.71	***	0.29	***	− 0.16	***
	Vegetables		0.60	***	− 1.11	***				
	Fruits^1^	Q2	2.71	***	− 3.15	**				
		Q3	3.86	***	− 4.82	***				
		Q4	5.82	***	− 10.16	***				
	Dried fruits and nuts^2^	Expenditure > 0	1.48	***	− 2.92	***				
Meat, fish, eggs			0.20	***	− 0.33	***				
	Red meat^1^	Q2	1.98	***	− 3.04	**	1.13	***	− 0.07	
		Q3	2.72	***	− 3.61	***	1.62	***	2.23	**
		Q4	3.84	***	− 9.72	***	2.84	***	1.41	*
	Processed meat^1^	Q2	0.02		2.39	**			2.10	**
		Q3	0.04		3.71	***			3.66	***
		Q4	− 0.30		6.49	***			7.26	***
	Eggs and poultry^1^	Q2	1.91	***	− 0.73		1.15	***		
		Q3	2.78	***	− 2.91	**	1.88	***		
		Q4	2.55	***	− 5.27	***	1.98	***		
	Fish^2^	0 < Expenditure ≤ median	3.01	***	− 1.92	*	2.26	***		
		Expenditure > median	6.49	***	− 7.01	***	5.20	***		
Starches^1^		Q2	0.45	^§^	− 2.20	**				
		Q3	− 0.05		− 4.91	***				
		Q4	− 1.30	***	− 12.37	***				
	Unrefined starches^2^	Expenditure > 0	3.30	***	− 7.51	***	2.05	***	− 4.17	***
	Refined grains^1^	Q2	− 0.60	*	0.96		− 0.31		− 2.92	***
		Q3	− 1.43	***	− 1.13		− 0.88	**	− 7.11	***
		Q4	− 3.28	***	− 7.76	***	− 2.20	***	− 14.12	***
Dairy products			0.01		0.76	***				
	Milk and yoghurt^1^	Q2	2.14	***	2.83	**	2.09	***	2.22	**
		Q3	2.57	***	5.26	***	3.22	***	3.29	***
		Q4	2.27	***	7.35	***	4.22	***	4.35	***
	Cheese^1^	Q2	1.49	***	2.02	**	1.02	***	3.30	***
		Q3	1.06	**	4.39	***	1.26	***	5.34	***
		Q4	− 0.23		8.67	***	0.82	**	10.37	***
Mixed dishes^2^		0 < Expenditure ≤ median	1.07	**	2.05	*	1.27	***	0.89	
		Expenditure > median	1.42	***	6.19	***	2.70	***	3.73	***
	Ready meals^2^	0 < Expenditure ≤ median	1.01	**	1.46	^§^				
		Expenditure > median	1.88	***	3.95	***				
	Savoury dishes^2^	Expenditure > 0	0.08		3.60	***				
Added fats^1^		Q2	0.44	^§^	− 1.60	^§^				
		Q3	− 0.63	^§^	− 0.82					
		Q4	− 4.26	***	− 2.50	**				
	Vegetable fats^2^	Expenditure > 0	0.63	**	− 9.35	***	− 0.49	**	− 6.69	***
	Animal fats^1^	Q2	0.20		1.50	^§^	− 0.49	*	1.63	**
		Q3	− 0.93	**	4.54	***	− 1.30	***	4.73	***
		Q4	− 4.26	***	9.77	***	− 3.86	***	9.35	***
Discretionary foods			− 0.26	***	1.19	***	− 0.13	***	1.19	***
	Savoury snacks^2^	Expenditure > 0	− 0.54	**	2.19	**				
	Sugar sweetened beverages^2^	Expenditure > 0	− 1.14	***	11.60	***				
	Calorie free beverages^2^	Expenditure > 0	− 0.18		3.07	***				
	Fruit juices^2^	Expenditure > 0	0.96	***	7.33	***				
	Sugared cereals^2^	Expenditure > 0	0.74	**	4.56	***				
	Dairy desserts^2^	Expenditure > 0	− 0.20		9.66	***				
	Sweet snacks^1^	Q2	− 0.12		11.55	***				
		Q3	− 2.81	***	17.10	***				
		Q4	− 6.24	***	25.66	***				
	Sauces^2^	Expenditure > 0	1.02	***	0.83					
Alcoholic beverages^2^		Expenditure > 0	− 0.93	***	− 6.11	***				

§*p* < 0.20; *p < 0.1; **p < 0.05; ***p < 0.001

^1^First quartile of expenditure share (Q1) as reference

^2^Non-purchasers (expenditure = 0) as reference

The results from the multivariate models can be seen in Table 2. All groups and subgroups that were included in the multivariate models were significantly associated with the MAR and/or the MER. As such, they can be considered as predictors of nutritional quality of FP, and were thus selected as potential subscore components. For the sake of simplification, efforts were made to reduce the number of subscore components. First, the “Mixed dishes” group was not included in the quality subscore since univariate regressions showed that it was both positively associated with the MAR and the MER. Moreover, removing the group from the multivariate analysis did not change the performance of the model (data not shown). This group is composed of dishes that are a combination of other groups (e.g. meat and vegetables) and is characterized by a high heterogeneity with dishes of both high and low nutritional quality. Second, the two “Starch” subgroups (unrefined starches and refined grains) were combined into a single variable by expressing the subscore component as the expenditure share of the unrefined starches subgroup within the starches group. Finally, two elements were combined in the “Fats” subscore component: “Total fats” and “Animal fats” expenditure shares within total food expenditures.

##### Definition of cut-offs for point allocation

The cut-offs and point allocation rules for each component of the subscores are summarized in Table 1. The segmented regression between the MAR and the “Fruits and Vegetables” expenditure shares showed two breakpoints at 6% and 23% of expenditures. As the regression slope between these two breakpoints is steep and a high proportion of households belong to this segment, deciles of expenditure shares were used to define intermediate cut-offs at 9% (3rd decile), 12% (median) and 16% (7th decile). Likewise, as the regression slope above 23% of expenditures is approximately zero, the allocation of additional points above this cut-off is not relevant. The “Fruits and Vegetables” component thus scored from 0 to 4 points across 4 cut-offs (6, 9, 12, 16%) as described in Table 1. Details of the methodology used and choices made for the definition of cut-offs of the other components are described in-depth in Supplemental Table S3. The “Cheese” component was scored from -1 to 1 across 2 cut-offs (4% and 8%). The “Milk and Yogurt” component scored 1 point for every expenditure share between 2 and 9%, 0.5 points when < 2% and 0 points when > 9%. The “Egg and Poultry” component scored 1 point for every expenditure share above 3%, and 0 points when < 3%. The “Fish” component scored from 0 to 2 points across 3 cut-offs (1.5%, 4%, 7%). The “Discretionary foods” component was scored from 0 to -3 points across 3 cut-offs (7%, 13%, 18%). The “Unrefined starches” component scored from 0 to 2 points across 3 cut-offs (0%, 18%, 30%). The “Red meat” component scored from 0 to -1 points across 1 cut-off (21%) classes. The “Processed meat” component scored from 0 to − 2 points across 2 cut-offs (6% and 10%).The “Fats” component scored from − 1 to 1 point across 2 cut-offs (1% and 2%).

The final version of the r-HPI (Table 1) is the sum of 15 components distributed amongst the diversity subscore (5 components) and the quality subscore (10 components). The total score can range between -8 and 17 points.

### Validity assessment of the r-HPI

**Fig. 1:**
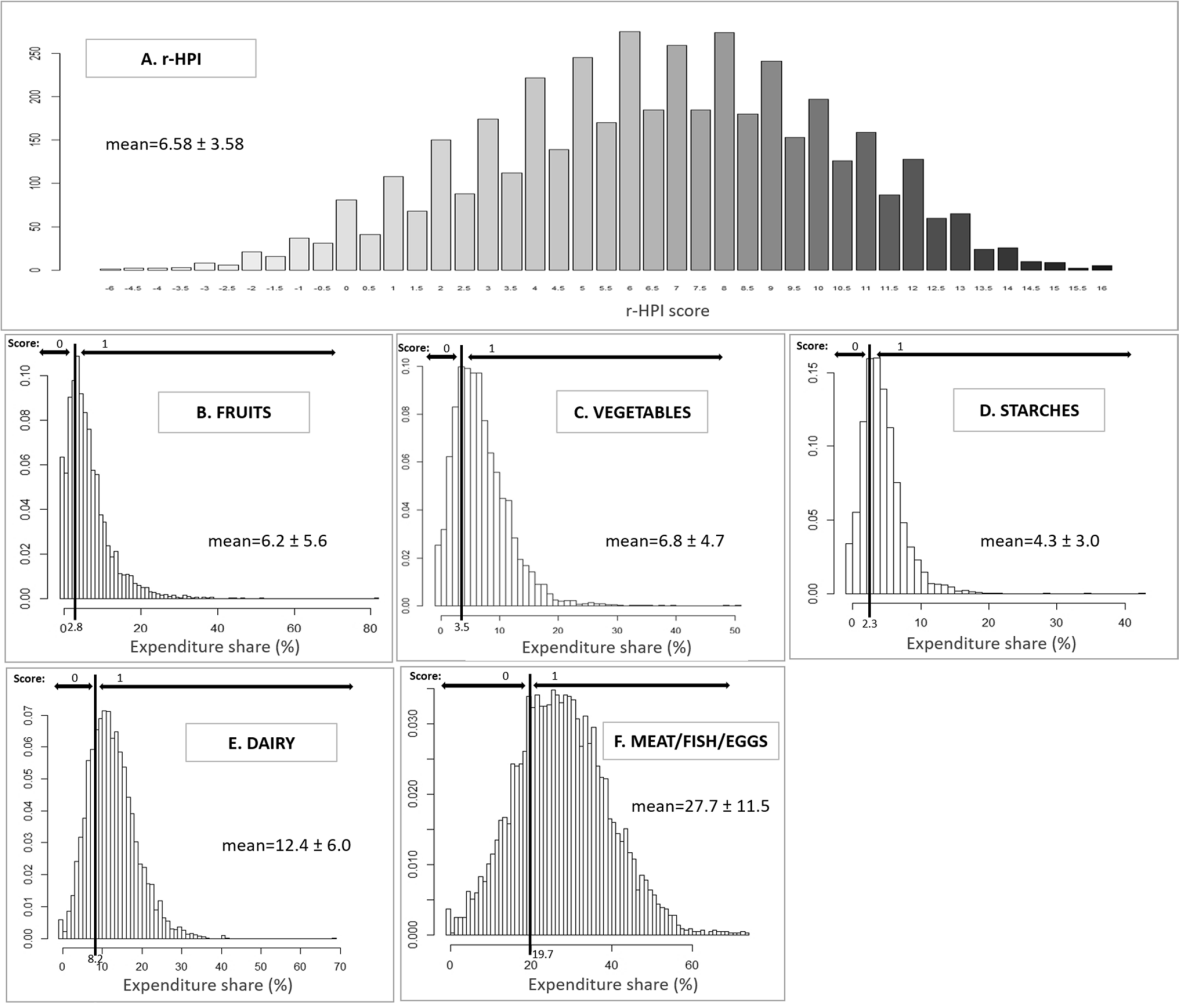
Distribution of the revised Healthy Purchase Index (r-HPI) (**A**) and of expenditure shares of household food purchases by component of the r-HPI diversity subscore (**B**–**F**) in the adaptation sample (*n* = 4375) of French households from the Kantar Worldpanel, respectively. “Score” mentioned on panels **B**–**F** refers to the number of points attributed according to cut-offs

**Fig. 2:**
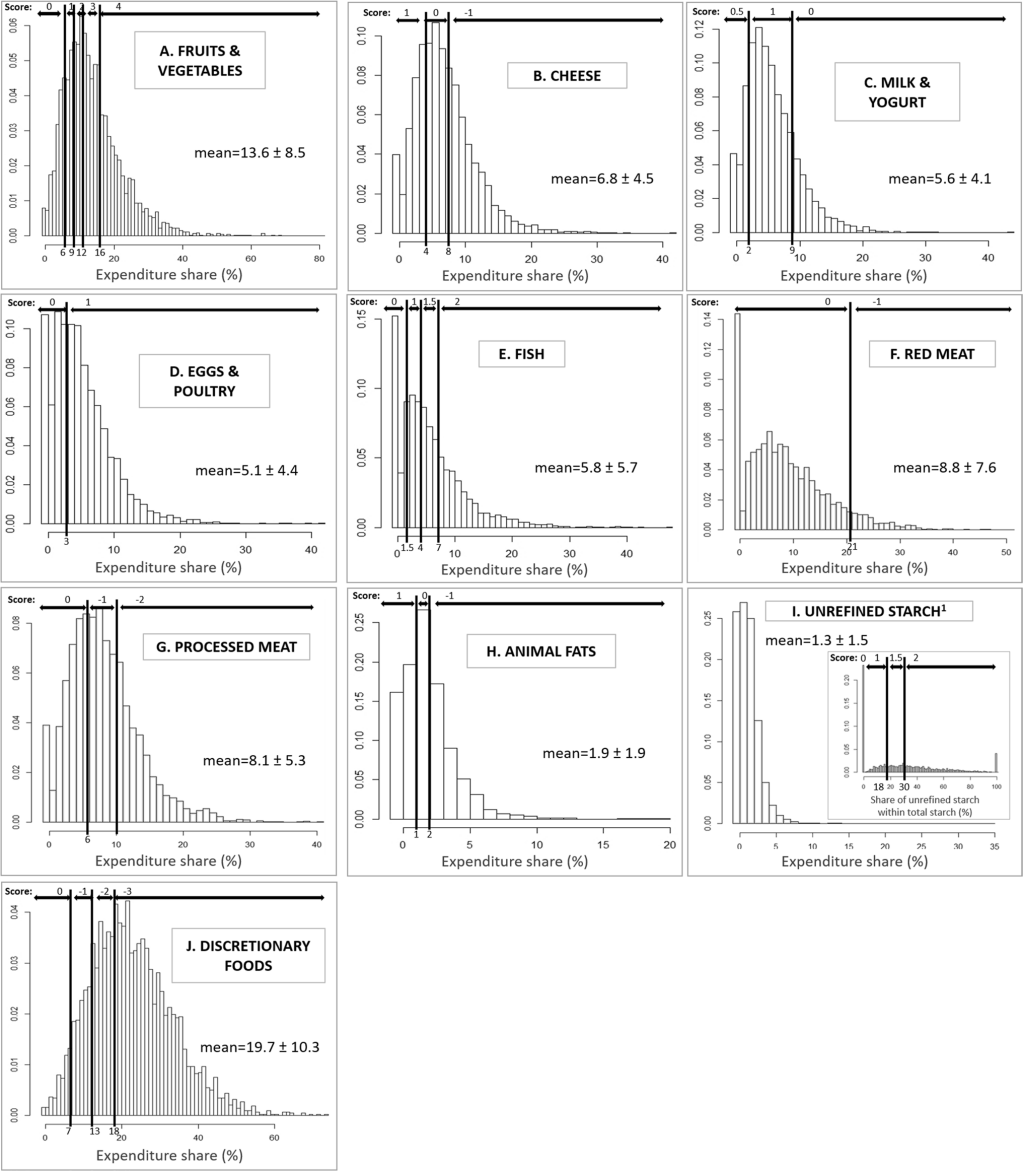
Distribution of the expenditure shares of household food purchases by component of the r-HPI nutritional quality subscore (**A**–**J**) in the adaptation sample (*n* = 4375) of French households from the Kantar Worldpanel, respectively. “Score” mentioned on the panels refers to the number of points attributed according to cut-offs. ^1^For the unrefined starch component, the cut-off values are based on the share of unrefined starch within total starch

The distributions of the r-HPI (Fig. 1A) and of expenditure shares of FP by component of the r-HPI in the 4375 households are presented in Figs. 1 and 2. The mean score was 6.58 ± 3.58, ranging from  −2 to 14 from the 1st to the 99th percentiles, respectively. Overall, the correlation between score components was low to moderate, mainly ranging from -0.300 and 0.350 (see Supplemental Table S4). The correlation between the r-HPI of FP and their total energy content was low (− 0.096, *p* < 0.001) (see Supplemental Table S4).

The r-HPI was significantly and positively correlated with the MAR and the NRF 9.3 and negatively with the SED, the MER and the excess ratios of SFA, free sugars and sodium (see Table 3).

**Table 3:** Correlations between the four nutritional quality indicators (NRF 9.3, MAR, MER, SED), excess ratios of nutrients included in the MER (SFA, free sugars, sodium) and the previous (HPI) and revised (r-HPI) version of the HPI applied to the food purchases of 4375 households from the French Kantar WorldPanel (adaptation sample)

	HPI (previous version)		r-HPI (revised version)	
NRF 9.3	0,61	***	0,60	***
MAR	0,51	***	0,53	***
MER	− 0,42	***	− 0,41	***
SED	− 0,66	***	− 0,65	***
SFA	− 0,16	***	− 0,27	***
Free sugars	− 0,39	***	− 0,29	***
Sodium	0,02		− 0,05	***

*HPI* Healthy Purchase Index, *MAR* mean adequacy ratio, *MER* mean excess ratio, *NRF 9.3* nutrient-rich foods index 9.3, *r-HPI* revised Healthy Purchase Index, *SED* solid energy density, *SFA* saturated fatty acids

*p < 0.1; **p < 0.05; ***p < 0.001

**Fig. 3:**
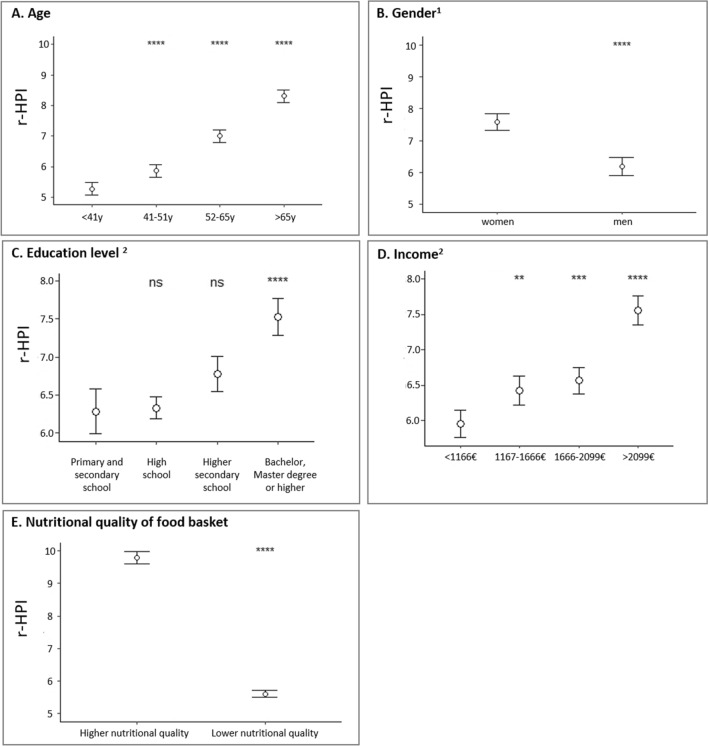
r-HPI of food purchases by age (**A**), gender (**B**), and education level (**C**) of the respondent, income per consumption unit (**D**), and for households having a food basket of higher (MAR > median and MER and SED < median) vs. lower nutritional quality (**E**), in the adaptation sample (*n* = 4375) of French households from the Kantar Worldpanel. *MAR* mean adequacy ratio, *MER* mean excess ratio, *r-HPI* revised Healthy Purchase Index, *SED* solid energy density. *p ≤ 0.05, **p ≤ 0.01, ***p ≤ 0.001; ****p ≤ 0.0001; ns: *p* > 0.05 (Wilcoxon test or pairwise comparisons of contrasts according to the tested variable); ^1^for gender, estimates and tests were performed on a subsample of single adult households (*n* = 1334); ^2^mean after adjustment for age

As shown in Fig. 3, the r-HPI of FP was significantly higher among households where the respondent was older (+2.92 between the oldest vs. youngest age group, *p* < 0.001), with higher education levels (+1.32 between the higher vs. lower education levels, *p* < 0.001), as well as for households with high income levels (+1.60 between the higher vs. lower quartile of income per consumption unit, *p* < 0.001) and single-adult households where the respondent was a woman (+ 1.41 vs. men, *p* < 0.001). A total of 1019 households were identified as having a food basket of higher nutritional quality within the population. The r-HPI of their FP was significantly higher (+ 4.15, *p* < 0.001) than households having a food basket of lower nutritional quality.

As regard external validity, similar results were obtained on the external validation sample of 2188 households (see Supplemental Figs. S1 and S2 and Supplemental Tables S4 and S5).

### Robustness assessment of the r-HPI

The associations between the r-HPI and the NRF 9.3, the MAR, the MER and the SED were statistically significant and of similar strength across the deciles of (i) total expenditure, (ii) alcoholic drinks expenditure shares, (iii) mixed dishes expenditure shares, and (iv) animal-to-plant protein ratio of the food basket in the adaptation sample (see Table 4). These results indicate that the r-HPI is efficient in assessing nutritional quality even for food baskets with extreme values of the studied characteristics. Similar results were obtained for the external validation sample (see Supplemental Table S6).

**Table 4:** Associations between the nutritional quality indicators (NRF 9.3, MAR, MER, SED) and the r-HPI of household food purchases across deciles of total expenditure (A), animal to plant protein ratio (B), “Alcoholic drinks” expenditure share (C), and “Mixed dishes” expenditure share (D) of the food basket, in the adaptation sample (*n* = 4375) of households from the French Kantar WorldPanel

(A) Decile of total expenditure (€)	NRF 9.3^1^	MAR^1^	MER^1^	SED^1^
D1: ≤ 111	0.555	0.470	− 0.384	− 0.577
D2:]111–147]	0.585	0.458	− 0.466	− 0.623
D3:]147–182]	0.614	0.513	− 0.410	− 0.640
D4:]182–217]	0.548	0.484	− 0.345	− 0.665
D5:]217–252]	0.552	0.488	− 0.347	− 0.651
D6:]252–285]	0.650	0.559	− 0.467	− 0.696
D7:]285–327]	0.640	0.534	− 0.490	− 0.684
D8:]327–385]	0.580	0.540	− 0.401	− 0.704
D9:]385–470]	0.592	0.517	− 0.389	− 0.686
D10: > 470	0.680	0.627	− 0.430	− 0.724

(B) Deciles of animal to plant protein ratio	NRF 9.3^1^	MAR^1^	MER^1^	SED^1^
D1: ≤ 1,63	0.597	0.490	− 0.402	− 0.595
D2:]1,63–2,04]	0.516	0.502	− 0.286	− 0.623
D3:]2,04–2,38]	0.635	0.604	− 0.386	− 0.703
D4:]2,38–2,68]	0.600	0.506	− 0.469	− 0.671
D5:]2,68–3,01]	0.601	0.562	− 0.429	− 0.671
D6:]3,01–3,40]	0.626	0.556	− 0.389	− 0.684
D7:]3,40–3,88]	0.619	0.576	− 0.447	− 0.695
D8:]3,88–4,58]	0.608	0.540	− 0.467	− 0.649
D9:]4,58–5,69]	0.572	0.497	− 0.439	− 0.634
D10: > 5,69	0.654	0.579	− 0.450	− 0.672

(C) Deciles of “Alcoholic drinks” expenditure share	NRF 9.3^1^	MAR^1^	MER^1^	SED^1^
D1: = 0	0.604	0.530	− 0.471	− 0.633
D2: = 0	0.604	0.530	− 0.471	− 0.633
D3: = 0	0.604	0.530	− 0.471	− 0.633
D4:]0–1,41]	0.531	0.528	− 0.332	− 0.670
D5:]1,41–3,15]	0.564	0.530	− 0.433	− 0.660
D6:]3,15–5,39]	0.632	0.595	− 0.474	− 0.683
D7:]5,39–8,36]	0.615	0.542	− 0.422	− 0.671
D8:]8,36–12,51]	0.602	0.504	− 0.442	− 0.682
D9:]12,51–20,26]	0.606	0.535	− 0.418	− 0.663
D10: > 20,26	0.595	0.464	− 0.357	− 0.611

(D) Deciles of “Mixed dishes” expenditure share	NRF 9.3^1^	MAR^1^	MER^1^	SED^1^
D1 = 0	0.548	0.471	− 0.436	− 0.618
D2 =]0–1.22]	0.672	0.580	− 0.515	− 0.654
D3 =]1.22–2.39]	0.589	0.528	− 0.379	− 0.631
D4 =]2.39–3.62]	0.606	0.540	− 0.418	− 0.703
D5 =]3.62–4.92]	0.596	0.522	− 0.387	− 0.670
D6 =]4.92–6.48]	0.616	0.537	− 0.394	− 0.640
D7 =]6.48–8.71]	0.590	0.590	− 0.367	− 0.661
D8 =]8.71–11.60]	0.565	0.511	− 0.390	− 0.658
D9 =]11.60–16.21]	0.617	0.569	− 0.401	− 0.687
D10 > 16.21	0.613	0.553	− 0.370	− 0.631

*MAR* mean adequacy ratio, *MER* mean excess ratio, *NRF 9.3* nutrient-rich foods index 9.3, *r-HPI* revised Healthy Purchase Index, *SED* solid energy density

^1^All associations were significant at *p* < 0.001

## Discussion

The present article details the adaptation for the general population and the validation of the r-HPI, a score that assesses the nutritional quality of FP. This index is comprised of two subscores (a diversity subscore and a quality subscore) and provides an assessment of the overall nutritional quality using the shares of food groups in total food expenditures, without requiring the pairing with a food composition table, nor information on the quantities purchased. We described the methodology used to revise the score components and cut-off values, and showed the concurrent validity and discriminating capacity of the r-HPI, based on a 4-week period of actual FP from a large sample of more than 4300 French households. Finally, external validity elements were provided by assessing the score’s performance on a second sample of more than 2100 French households, and robustness analyses showed that the r-HPI is efficient in reflecting the nutritional quality of household FP with varying characteristics in terms of total expenditures or animal-to-plant protein ratio in particular.

The present study evaluated at length the validity of the r-HPI by assessing its concurrent validity, discriminating capacity and external validity. The results showed a good correlation between the r-HPI and each of the four indicators of nutritional quality (i.e. NRF 9.3, MAR, MER, SED). Additionally, the evaluation corroborated the fact that the r-HPI differentiates groups based on criteria that are knowingly related to diet quality (age, gender, income and education level), and gave higher scores to households whose food basket was classified of higher nutritional quality within the population. The r-HPI also showed good validity across the various levels of total food expenditures, as evaluated against the four indicators of nutritional quality. In addition, the validity of the r-HPI was likewise supported by a large variability in scores among the studied population, a low correlation between score components (showing the relevance of including all components in the score), and a low correlation between the total score and energy content of FP (indicating that the score is able to reflect the nutritional quality of food purchases independently of the quantities purchased). Finally, similar results were obtained for the whole set of validity analyses performed on a second sample of more than 2,100 households (whose purchase data was not used to adapt the score), hence demonstrating the external validity of the r-HPI.

Despite the great potential of FP data to study dietary behaviours, their use remains especially limited for nutritional quality assessment, due to the labour involved in data processing, such as food item data entry and pairing of items with the food composition table. The r-HPI has the core advantage of overcoming these barriers and facilitating FP data assessment as it only requires the expenditure shares of broad food groups and not food item quantity or nutrient content.

Moreover, the revised version of the HPI described in the present study was developed from real food expenditures of more than 4300 French households, covering a large diversity of FP patterns and socio-demographic characteristics. In addition, FP data used for adaptation and validation of the r-HPI cover all types of food outlets frequented by panellists (except bakeries), and include fresh products. To our knowledge, only two other indexes have been developed to assess the nutritional quality of FP using expenditure shares of food groups: the Grocery Purchase Quality Index-2016 (GPQI-2016) [28], and the Healthy Trolley Index (HETI) [29]. However, the GPQI-2016 was developed based on the expenditure shares of the USDA Food Plan’s market baskets—designed for the US population—where scoring signifies the degree of adherence to US Dietary Guidelines. Hence, this score is based on theoretical food baskets whose cultural acceptability remains questionable. Regarding the HETI score, it is computed based on a direct comparison of food group expenditure shares with a benchmark cut-off calculated as the percentage of servings per food group in total daily servings recommended in the Australian dietary guidelines. It is likely that the weight share of a given food group may not accurately reflect expenditure shares because some food groups are more expensive than others [30]. This direct computation can thus question the relevance of the HETI scoring system. Unlike the HETI, the benchmark cut-offs used to compute the r-HPI are directly expressed in food group expenditure shares.

Beyond health issues, the major environmental impact of current food systems [31, 32] needs to be mitigated by actions on food production, transformation and waste combined with shifts in food choices towards more sustainable diets [33], defined as diets with low environmental impacts, nutritionally adequate, culturally acceptable, accessible, economically fair and affordable, safe and healthy [34]. Animal-based products, in particular red meat, have been identified as a key component in reducing the environmental impact of diets [35]. Therefore, on top of its adaptation to the general population, a strength of the r-HPI vs. the HPI is that attention has been paid to explicitly consider “Red meat” and “Processed meat” subgroups, which may prove useful when assessing the sustainability of FP. Robustness analyses additionally ensure that the performance of the r-HPI is maintained across different animal-to-plant protein ratios in the food baskets.

The main interest of the r-HPI lies in its application to various domains. First, the r-HPI is a valuable tool for studying socioeconomic disparities in diet quality because it is based on real food expenditures. Most studies assessing the relationships between dietary quality and diet costs are performed by estimating the cost of individual diets based on mean food prices [6], as though all individuals in a given population are purchasing foods at exactly the same price, which is not the case. Second, considering the increasing concern in the role of environmental factors in food and health behaviours [36], the r-HPI allows for the exploration of relationships between real FP, food outlet frequentation and the food environment, defined as the physical, organizational and sociocultural space in which people encounter meals and food [3]. Third, the r-HPI could be used to explore the sustainability of food systems. In particular, it can easily be used by researchers of disciplines other than nutrition to apply a nutritional quality indicator without requiring to deal with food composition tables. Finally, the food expenditure cut-offs of the r-HPI can be used as a practical tool in health promotion to provide recommendations to improve the nutritional quality of FP, in particular to disadvantaged households with budget constraints.

Nonetheless, the present study has limitations. First, although FP data used for adaptation and validation of the r-HPI include various types of food sources and include fresh products, they do not include food and beverages purchased from restaurants and thus do not reflect consumption outside of the home. This being said, in France [37] and on a larger scale in Europe [38], the majority of meals are primarily consumed at home: out-of-home consumption accounts for 22% of total energy intake of French adults, and between 19 and 24% of total intakes for all minerals and vitamins [37]. Although home consumption represents a large share of total intakes, and some studies suggest that purchase data are able to describe food consumption in an adult population [39], it is important to bear in mind that nutritional quality can differ depending on the place (home vs. out-of-home) of consumption. Second, it should be noted that the r-HPI assesses the nutritional quality of FP at the household level without distinguishing subsequent allocation of foods between household members, and should thus not be considered as an indicator of individual diet quality. Finally, the r-HPI was developed from FP of a French population and may therefore not be directly applicable to other populations. However, the methods used for its development and validation can easily be reproduced with FP data from other countries.

## Conclusion

The present study is based on real FP data from a large sample of French households and provides evidence to support the concurrent validity, discriminating capacity and external validity of the r-HPI, a score that assesses the nutritional quality of FP based on expenditure shares of specific food groups. As the r-HPI does not require information on the quantity or nutrient content of food items, this score is a valuable tool that will facilitate the use of FP data in the exploration of sustainability of food behaviours, in particular with regard to their relationship with the food environment, or to evaluate the impact of interventions targeting dietary behaviours. In addition, food expenditure cut-off values that were determined in the r-HPI score can be used in health promotion to provide nutri-economic counselling, especially for households under budgetary constraints.

## Supplementary Information

Below is the link to the electronic supplementary material.

Supplementary file1 (DOCX 220 KB)
