# Supplementary material for: The revised Healthy Purchase Index (r-HPI): a validated tool for exploring the nutritional quality of household food purchases
Source: Eur J Nutr. 2022 Aug 27;62(1):363–77. doi: 10.1007/s00394-022-02962-4 (PMC9899728; doi:10.1007/s00394-022-02962-4)
Supplement: Supplementary file 1 — Supplementary file1 (DOCX 220 KB) [file 394_2022_2962_MOESM1_ESM.docx]

**Supplementary Information**

**The revised Healthy Purchase Index (r-HPI): a validated tool for exploring the nutritional quality of household food purchases**

European Journal of Nutrition

Marlène Perignon^1^, Pascaline Rollet^1^, Marion Tharrey^1^, Daisy Recchia^1^, Sophie Drogué^1^, France Caillavet^2^, Caroline Mejean^1^, Nicole Darmon^1^

^1^ MoISA, Univ Montpellier, CIHEAM-IAMM, CIRAD, INRAE, Institut Agro, IRD, Montpellier, France

^2^ ALISS, INRAE, Univ Paris-Saclay, Ivry, France

Corresponding author: Marlène Perignon, [marlene.perignon@inrae.fr](mailto:marlene.perignon@inrae.fr)

**Supplemental Table S1**: Food groups and subgroups classification of food items purchased by households from the Kantar Worldpanel 2014

| **Groups** | **Subgroups** | **Examples of food items included** |
| --- | --- | --- |
| **Fruits & Vegetables** | **Vegetables** | Fresh vegetables, vegetable soup, canned vegetables |
|  | **Fruits** | Fresh fruit, canned fruit, stewed fruit |
|  | **Dried fruits & nuts** | Unsalted dried fruit, nuts, seeds |
| **Meat, fish, eggs** | **Red meat** | Beef, pork, lamb, |
|  | **Processed meat** | Cured and cooked ham, sausages, bacon, pâté |
|  | **Eggs & poultry** | Hard-boiled egg, fried egg, omelette, chicken, duck, turkey |
|  | **Fish** | Fresh fish, canned fish, shellfish, surimi |
| **Starches** | **Unrefined starches** | Potatoes, legumes, wholegrain products |
|  | **Refined grains** | Bread rolls, fresh bread, pasta, rice, flour |
| **Dairy products** | **Milk & yoghurt** | Refrigerated and long-life milk, plain yoghurt, sweetened yoghurt, fruit yoghurt, yoghurt drink |
|  | **Cheese** | Hard cheese, soft cheese, cream cheese |
| **Mixed dishes** | **Ready meals** | (frozen) ready meals, canned meals, salads |
|  | **Savoury dishes** | Quiche, pizza, savoury pies, puff pastries, burgers, sandwiches |
| **Added fats** | **Vegetable fats** | Vegetable oil, margarine, salad dressing |
|  | **Animal fats** | Cream, butter |
| **Discretionary foods** | **Savoury snacks** | crackers, chips, salted and roasted nuts, olives |
|  | **Sugar sweetened beverages** | Soda, nectars |
|  | **Calorie free beverages** | Diet soft drinks |
|  | **Fruit juices** | Fresh fruit juice, concentrated fruit juice |
|  | **Sugared cereals** | Breakfast cereals |
|  | **Dairy desserts** | Cream dessert, ice cream |
|  | **Sweet snacks** | Cakes, biscuits, pastries, candies, chocolate |
|  | **Sauces** | Ketchup, sauces including soya/tomato/barbecue, etc. |
| **Alcoholic beverages** | **Alcoholic beverages** | Beer, wine, liquor, whiskey |
| **Condiments** | **Spices** | Salt, pepper, herbs, spices, mustard, pickles |
| **Waters and hot beverages** | **Waters and hot beverages** | Mineral water, sparkling water, tea, coffee |
| **Baby foods** | **Baby foods** | Infant formulas, baby food jars |

**Supplemental Table S2**: Socio-economic and food purchases characteristics in the adaptation sample (n=4,375 households) and the external validation sample (n=2,188 households)

|  |  | **Adaptation sample (n=4375)** | | **External validation sample (n=2188)** | |  |
| --- | --- | --- | --- | --- | --- | --- |
|  |  | Mean^1^ | SD | Mean^1^ | SD | p-value^2^ |
| **Age of the respondent (y)** | | 52.6 | 15.3 | 53.1 | 15.4 | 0.17 |
| **Number of household members** | | 2.475 | 1.35 | 2.441 | 1.39 | 0.15 |
| **Income per consumption unit (€/month)** | | 1748.5 | 809.3 | 1711.7 | 786.4 | 0.07 |
| **Household structure** | |  |  |  |  | 0.10 |
|  | 1 adult | 30.49% |  | 32.13% |  |  |
|  | 1 adult with child(ren) | 1.87% |  | 1.14% |  |  |
|  | Several adults | 41.1% |  | 40.49% |  |  |
|  | Several adults with child(ren) | 26.54% |  | 26.23% |  |  |
| **Level of education of the respondent** | |  |  |  |  | 0.75 |
|  | Primary and secondary school | 13.04% |  | 13.88% |  |  |
|  | High school | 48.98% |  | 48.96% |  |  |
|  | Higher secondary school | 19.8% |  | 19.01% |  |  |
|  | Bachelor or Master degree or higher | 18.18% |  | 18.14% |  |  |
| **Socio-professional category of the respondent** | |  |  |  |  | 0.85 |
|  | Farmers | 0.25% |  | 0.23% |  |  |
|  | Artisans. shopkeepers. entrepreneurs | 1.46% |  | 1.14% |  |  |
|  | Executives and intellectual professionals | 6.67% |  | 6.35% |  |  |
|  | Intermediate professions | 15.45% |  | 14.44% |  |  |
|  | Employees | 34.35% |  | 34.6% |  |  |
|  | Workers | 4.37% |  | 4.25% |  |  |
|  | Retired | 28.11% |  | 29.34% |  |  |
|  | Unemployed | 9.33% |  | 9.64% |  |  |
| **Residence area** | |  |  |  |  | 0.76 |
|  | Paris region | 18.1% |  | 18.37% |  |  |
|  | Greater Paris Area | 18.51% |  | 18.83% |  |  |
|  | North | 7.34% |  | 6.54% |  |  |
|  | East | 9.67% |  | 9.73% |  |  |
|  | West | 15.41% |  | 16.73% |  |  |
|  | South-West | 10.72% |  | 10.42% |  |  |
|  | Central East | 10.49% |  | 10.42% |  |  |
|  | Mediterranean area | 9.76% |  | 8.96% |  |  |
| **Food expenditure (€/4 weeks)** | | 298.7 | 160.9 | 292.7 | 157.6 | 0.13 |
| **Food expenditure per person (€/4 weeks)** | | 142.2 | 84.1 | 142.2 | 80.8 | 0.88 |
| ^1^or % where specified, ^2^Wilcoxon tests (for age, number of household members, income, food expenditure) or Chi² tests (for household structure, level of education, socio-professional category, residence area) between adaptation and external validation samples characteristics | | | | | | |

**Supplemental Table 3**: definition of cut-off values for point allocation

| **Subscore component** | **Definition of cut-off values** |
| --- | --- |
| **Fruits & Vegetables** | The segmented regression between the MAR and the “Fruits & Vegetables” expenditure share showed two breakpoints at 6% and 23% of expenditure. The regression slope between these two breakpoints being steep and a high proportion of households belonging to this segment, deciles of expenditure share were used to define intermediate cut-offs at 9% (3^rd^ decile), 12% (median) and 16% (7^th^ decile). The regression slope above 23% of expenditure being approximately null, attribution of additional point above this cut-off was not relevant. The “Fruits & Vegetables” component thus scored from 0 to 4 points across 4 cut-offs (6%, 9%, 12%, 16%) as described in **Table 1**. |
| **Cheese** | The segmented regression between the MAR and the “Cheese” subgroup expenditure share showed a breakpoint at 4% of expenditure. Moreover, higher values of MER being observed for expenditure share above 5%, deciles were used to define an additional cut-off at 8% (7^th^ decile) above which the MAR was lower and the MER was higher. The “Cheese” component thus scored from -1 to 1 across 2 cut-offs (4% and 8%) as described in **Table 1**. |
| **Milk & Yogurt** | The segmented regression between the MAR and the “Milk & Yogurt” subgroup expenditure share showed 2 breakpoints at 2% and 9% of expenditure, with a positive slope between 0-2%, a stabilization between 2-9%, and a negative slope above 9%. Moreover, the segmented regression between the MER and the “Milk & Yogurt” subgroup expenditure share identified a breakpoint at 2% of expenditure, with positive slopes between 0-2% and above 2%, indicating a lower nutritional quality for an expenditure share above 9% (hence a lower number of point) than below 2%. The “Milk & Yogurt” component thus scored 1 point for an expenditure share between 2-9%, 0.5 point when <2% and 0 point when >9%. |
| **Egg & Poultry** | The segmented regression between the MAR and the “Egg & Poultry” subgroup expenditure share showed a breakpoint at 3%. This component thus scored 1 point for an expenditure share above 3%, and 0 point when <3%. |
| **Fish** | The segmented regression between the MAR and the “Fish” subgroup expenditure share showed 2 breakpoints at 1.5% and 9%. The slope between these two breakpoints being steep and a high proportion of households belonging to this segment, deciles of expenditure share were used to define intermediate cut-offs at 4% (median). In addition, the fish consumption guidelines being limited to 2 times / week due to a risk of exposure to food contaminants, the maximum cut-off was lowered from 9% to 7% (7th decile) to avoid encouraging a too high consumption. The “Fish” component thus scored from 0 to 2 points across 3 cut-offs (1.5%, 4% and 7%) as described in **Table 1**. |
| **Discretionary foods** | The segmented regressions between the “Discretionary foods” group expenditure share and the MAR and the MER both showed a breakpoint at 13% of expenditure. The MER regression slopes being steep between 0-13% and 13-21%, and a high proportion of households belonging to these segments, deciles of expenditure share were used to define intermediate cut-offs at 7% (1^st^ decile) and 18% (median). The “Discretionary foods” component thus scored from 0 to -3 points across 3 cut-offs (7%, 13% and 18%) as described in **Table 1**. |
| **Unrefined starches** | The segmented regressions between the MAR and the “Unrefined starches” subgroup expenditure share within starches group showed breakpoints at 30% of expenditure. The regression slope being steep between 0-30% and a high proportion of households belonging to this segment (60% of the sample has an expenditure share <32%), deciles of expenditure share were used to define an intermediate cut-off at 18% (4th decile). The “Unrefined starches” component thus scored from 0 to 2 points across 4 classes as described in **Table 1**. |
| **Red meat** | The segmented regression between the MAR and the “Red meat” subgroup expenditure share showed a breakpoint at 21% of expenditure, with an inversion of slope that became negative – hence an impairment of nutritional quality – for expenditure share >21%. Considering that guidelines are to limit red meat consumption, this component scored from 0 to -1 points across 2 classes as described in **Table 1**. |
| **Processed meat** | The segmented regression between the MER and the “Processed meat” subgroup expenditure share showed a breakpoint at 6% of expenditure. Deciles were used to add an additional cut-off at 10% (7^th^ decile). This component thus scored from 0 to -2 points across 3 classes as described in **Table 1**. |
| **Fats** | For the “Fats” group, households with no purchase of fats had a lower MAR and a higher MER than households purchasing fats but 0% of animal fats, hence 0 point was attributed in the first case and 1 point for the second. MAR decreased and MER increased with higher expenditure share of animal fats: deciles of expenditure share were used to define additional cut-offs at 1% (3^rd^ decile=0.8%) and 2% (6^th^ decile=1.9%). The “animal fats” component thus scored from -1 to 1 point as described in **Table 1**. |

MAR: Mean Adequacy Ratio; MER: Mean Excess Ratio

**Supplemental Table S4**: Correlations between the revised Healthy Purchase Index (r-HPI) components, total score and energy content of food purchases, in the adaptation sample (panel A, n=4,375) and the external validation sample (panel B, n=2,188) of French households from the Kantar Worldpanel

(A)

|  |  | Fruits | | Vegetables | | | Starches | | | Dairy | | | MFE | | | FV | | Cheese | | | Milk & Yogurts | | | Eggs & Poultry | | | Fish | | | Red meat | | | Processed meat | | Fats | | Unrefined starch | | Discretionary foods | | r-HPI | | Energy |
| --- | --- | --- | --- | --- | --- | --- | --- | --- | --- | --- | --- | --- | --- | --- | --- | --- | --- | --- | --- | --- | --- | --- | --- | --- | --- | --- | --- | --- | --- | --- | --- | --- | --- | --- | --- | --- | --- | --- | --- | --- | --- | --- | --- |
| Diversity subscore | Fruits | 1.000 |  | |  |  | |  |  | |  |  | |  |  |  |  | |  |  | |  |  | |  |  | |  |  | |  |  |  |  |  |  |  |  |  |  |  |  |  |
|  | Vegetables | 0.224 | *** | | 1.000 |  | |  |  | |  |  | |  |  |  |  | |  |  | |  |  | |  |  | |  |  | |  |  |  |  |  |  |  |  |  |  |  |  |  |
|  | Starches | 0.013 |  | | 0.083 | *** | | 1.000 |  | |  |  | |  |  |  |  | |  |  | |  |  | |  |  | |  |  | |  |  |  |  |  |  |  |  |  |  |  |  |  |
|  | Dairy | 0.021 |  | | 0.031 | * | | 0.097 | *** | | 1.000 |  | |  |  |  |  | |  |  | |  |  | |  |  | |  |  | |  |  |  |  |  |  |  |  |  |  |  |  |  |
|  | MFE | 0.030 | * | | 0.072 | *** | | 0.005 |  | | -0.052 | *** | | 1.000 |  |  |  | |  |  | |  |  | |  |  | |  |  | |  |  |  |  |  |  |  |  |  |  |  |  |  |
| Quality subscore | FV | 0.598 | *** | | 0.533 | *** | | 0.039 | * | | -0.003 |  | | 0.017 |  | 1.000 |  | |  |  | |  |  | |  |  | |  |  | |  |  |  |  |  |  |  |  |  |  |  |  |  |
|  | Cheese | 0.021 |  | | -0.006 |  | | -0.073 | *** | | -0.525 | *** | | 0.020 |  | 0.034 | * | | 1.000 |  | |  |  | |  |  | |  |  | |  |  |  |  |  |  |  |  |  |  |  |  |  |
|  | Milk & Yogurts | 0.042 | ** | | 0.036 | * | | 0.016 |  | | 0.019 |  | | 0.105 | *** | 0.017 |  | | -0.055 | *** | | 1.000 |  | |  |  | |  |  | |  |  |  |  |  |  |  |  |  |  |  |  |  |
|  | Eggs & Poultry | 0.017 |  | | 0.043 | ** | | 0.084 | *** | | -0.008 |  | | 0.342 | *** | -0.005 |  | | -0.003 |  | | 0.077 | *** | | 1.000 |  | |  |  | |  |  |  |  |  |  |  |  |  |  |  |  |  |
|  | Fish | 0.119 | *** | | 0.101 | *** | | -0.046 | ** | | -0.068 | *** | | 0.300 | *** | 0.133 | *** | | 0.058 | *** | | 0.052 | *** | | 0.027 |  | | 1.000 |  | |  |  |  |  |  |  |  |  |  |  |  |  |  |
|  | Red meat | -0.004 |  | | -0.030 | * | | 0.067 | *** | | 0.099 | *** | | -0.164 | *** | 0.018 |  | | -0.086 | *** | | -0.031 | * | | 0.006 |  | | 0.019 |  | | 1.000 |  |  |  |  |  |  |  |  |  |  |  |  |
|  | Processed meat | 0.078 | *** | | 0.059 | *** | | -0.028 |  | | -0.041 | ** | | -0.301 | *** | 0.122 | *** | | 0.086 | *** | | -0.013 |  | | -0.052 | *** | | 0.088 | *** | | -0.020 |  | 1.000 |  |  |  |  |  |  |  |  |  |  |
|  | Fats | 0.026 |  | | 0.012 |  | | -0.066 | *** | | -0.090 | *** | | -0.016 |  | 0.029 |  | | 0.081 | *** | | 0.009 |  | | -0.043 | ** | | 0.041 | ** | | -0.016 |  | 0.080 | *** | 1.000 |  |  |  |  |  |  |  |  |
|  | Unrefined starch | 0.078 | *** | | 0.156 | *** | | 0.182 | *** | | -0.004 |  | | 0.097 | *** | 0.142 | *** | | -0.015 |  | | 0.061 | *** | | 0.058 | *** | | 0.021 |  | | -0.043 | ** | 0.013 |  | 0.062 | *** | 1.000 |  |  |  |  |  |  |
|  | Discretionary foods | 0.137 | *** | | 0.140 | *** | | -0.102 | *** | | -0.095 | *** | | 0.201 | *** | 0.242 | *** | | 0.002 |  | | -0.004 |  | | 0.064 | *** | | 0.201 | *** | | -0.200 | *** | -0.034 | * | 0.020 |  | 0.107 | *** | 1.000 |  |  |  |  |
| Total score | r-HPI | 0.518 | *** | | 0.508 | *** | | 0.150 | *** | | -0.051 | *** | | 0.246 | *** | 0.722 | *** | | 0.192 | *** | | 0.151 | *** | | 0.211 | *** | | 0.417 | *** | | -0.016 |  | 0.282 | *** | 0.272 | *** | 0.395 | *** | 0.465 | *** | 1.000 |  |  |
|  | Energy | -0.092 | *** | | -0.017 |  | | 0.201 | *** | | 0.098 | *** | | 0.157 | *** | -0.223 | *** | | -0.071 | *** | | 0.147 | *** | | 0.175 | *** | | -0.019 |  | | 0.009 |  | -0.079 | *** | -0.065 | *** | 0.077 | *** | -0.222 | *** | -0.096 | *** | 1.000 |

* p< 0.05; **p< 0.01; ***p< 0.001 ; FV : Fruits and Vegetables, MFE: Meat/Fish/eggs; r-HPI: revised Healthy Purchase Index

(B)

|  |  | Fruits | | Vegetables | | Starches | | Dairy | | MFE | | FV | | Cheese | | Milk & Yogurts | | Eggs & Poultry | | Fish | | Red meat | | | Processed meat | | | Fats | | | Unrefined starch | | | Discretionary foods | | | r-HPI | | | Energy | |
| --- | --- | --- | --- | --- | --- | --- | --- | --- | --- | --- | --- | --- | --- | --- | --- | --- | --- | --- | --- | --- | --- | --- | --- | --- | --- | --- | --- | --- | --- | --- | --- | --- | --- | --- | --- | --- | --- | --- | --- | --- | --- |
| Diversity subscore | Fruits | 1.000 |  |  |  |  |  |  |  |  |  |  |  |  |  |  |  |  |  |  |  |  |  |  | |  |  | |  |  | |  |  | |  |  | |  |  | |  |
|  | Vegetables | 0.226 | *** | 1.000 |  |  |  |  |  |  |  |  |  |  |  |  |  |  |  |  |  |  |  |  | |  |  | |  |  | |  |  | |  |  | |  |  | |  |
|  | Starches | 0.001 |  | 0.090 | *** | 1.000 |  |  |  |  |  |  |  |  |  |  |  |  |  |  |  |  |  |  | |  |  | |  |  | |  |  | |  |  | |  |  | |  |
|  | Dairy | 0.040 |  | 0.034 |  | 0.120 | *** | 1.000 |  |  |  |  |  |  |  |  |  |  |  |  |  |  |  |  | |  |  | |  |  | |  |  | |  |  | |  |  | |  |
|  | MFE | 0.031 |  | 0.074 | *** | 0.008 |  | -0.048 | * | 1.000 |  |  |  |  |  |  |  |  |  |  |  |  |  |  | |  |  | |  |  | |  |  | |  |  | |  |  | |  |
| Quality subscore | FV | 0.601 | *** | 0.580 | *** | 0.013 |  | 0.030 |  | -0.007 |  | 1.000 |  |  |  |  |  |  |  |  |  |  |  |  | |  |  | |  |  | |  |  | |  |  | |  |  | |  |
|  | Cheese | -0.009 |  | 0.008 |  | -0.076 | *** | -0.529 | *** | 0.006 |  | 0.013 |  | 1.000 |  |  |  |  |  |  |  |  |  |  | |  |  | |  |  | |  |  | |  |  | |  |  | |  |
|  | Milk & Yogurts | 0.042 | * | 0.043 | * | 0.024 |  | -0.003 |  | 0.131 | *** | 0.047 | * | -0.034 |  | 1.000 |  |  |  |  |  |  |  |  | |  |  | |  |  | |  |  | |  |  | |  |  | |  |
|  | Eggs & Poultry | 0.020 |  | 0.077 | *** | 0.053 | * | 0.035 |  | 0.280 | *** | 0.022 |  | -0.004 |  | 0.072 | *** | 1.000 |  |  |  |  |  |  | |  |  | |  |  | |  |  | |  |  | |  |  | |  |
|  | Fish | 0.124 | *** | 0.109 | *** | -0.025 |  | -0.106 | *** | 0.304 | *** | 0.124 | *** | 0.071 | *** | 0.045 | * | -0.007 |  | 1.000 |  |  |  |  | |  |  | |  |  | |  |  | |  |  | |  |  | |  |
|  | Red meat | 0.014 |  | -0.010 |  | 0.019 |  | 0.082 | *** | -0.160 | *** | 0.004 |  | -0.075 | *** | -0.019 |  | 0.008 |  | -0.002 |  | 1.000 |  |  | |  |  | |  |  | |  |  | |  |  | |  |  | |  |
|  | Processed meat | 0.045 | * | 0.056 | ** | -0.017 |  | -0.031 |  | -0.330 | *** | 0.133 | *** | 0.089 | *** | -0.069 | ** | -0.046 | * | 0.055 | ** | -0.009 |  | 1.000 | |  |  | |  |  | |  |  | |  |  | |  |  | |  |
|  | Fats | -0.018 |  | -0.001 |  | -0.092 | *** | -0.082 | *** | -0.030 |  | 0.013 |  | 0.079 | *** | 0.042 |  | -0.024 |  | 0.045 | * | 0.010 |  | 0.067 | | ** | 1.000 | |  |  | |  |  | |  |  | |  |  | |  |
|  | Unrefined starch | 0.023 |  | 0.148 | *** | 0.167 | *** | -0.042 | * | 0.077 | *** | 0.108 | *** | 0.034 |  | 0.073 | *** | 0.038 |  | 0.004 |  | -0.041 |  | 0.003 | |  | 0.014 | |  | 1.000 | |  |  | |  |  | |  |  | |  |
|  | Discretionary foods | 0.140 | *** | 0.178 | *** | -0.090 | *** | -0.090 | *** | 0.200 | *** | 0.270 | *** | 0.009 |  | 0.002 |  | 0.013 |  | 0.176 | *** | -0.171 | *** | -0.065 | | ** | 0.013 | |  | 0.134 | | *** | 1.000 | |  |  | |  |  | |  |
| Total score | r-HPI | 0.491 | *** | 0.563 | *** | 0.146 | *** | -0.039 |  | 0.216 | *** | 0.743 | *** | 0.197 | *** | 0.164 | *** | 0.197 | *** | 0.392 | *** | -0.018 |  | 0.262 | | *** | 0.249 | | *** | 0.368 | | *** | 0.470 | | *** | 1.000 | |  |  | |  |
|  | Energy | -0.054 | * | -0.054 | * | 0.203 | *** | 0.098 | *** | 0.084 | *** | -0.213 | *** | -0.090 | *** | 0.086 | *** | 0.173 | *** | -0.007 |  | 0.056 | ** | -0.076 | | *** | -0.071 | | *** | 0.060 | | ** | -0.256 | | *** | -0.124 | | *** | 1.000 | |  |

* p< 0.05; **p< 0.01; ***p< 0.001 ; FV : Fruits and Vegetables, MFE: Meat/Fish/eggs; r-HPI: revised Healthy Purchase Index

**Supplemental Table S5**: Correlations between the four nutritional quality indicators (NRF 9.3, MAR, MER, SED), excess ratios of nutrient included in the MER (SFA, free sugars, sodium) and the previous (HPI) and revised (r-HPI) version of the HPI in the external validation sample of 2,188 households from the French Kantar WorldPanel.

|  | **HPI**  **(previous version)** | | **r-HPI**  **(revised version)** | |
| --- | --- | --- | --- | --- |
| **NRF 9.3** | 0,62 | *** | 0.60 | *** |
| **MAR** | 0,51 | *** | 0.53 | *** |
| **MER** | -0,42 | *** | -0.40 | *** |
| **SED** | -0,69 | *** | -0.67 | *** |
| **SFA** | -0,14 | *** | -0.23 | *** |
| **Free sugars** | -0,40 | *** | -0.32 | *** |
| **Sodium** | 0,04 | ** | -0.01 |  |

* p <0.1; ** p <0.05; *** p<0.001

**Supplemental Table S6**: Associations between the nutritional quality indicators (NRF 9.3, MAR, MER, SED) and the r-HPI of household food purchases across deciles of total expenditure (A), animal to plant protein ratio (B), “Alcoholic drinks” expenditure share (C), and “Mixed dishes” expenditure share (D) of the food basket, in the external validation sample (n=2,188) of households from the Kantar WorldPanel

| **(A) Decile of total expenditure (€)** | **NRF 9.3^1^** | **MAR^1^** | **MER^1^** | **SED^1^** |
| --- | --- | --- | --- | --- |
| **D1 : ≤ 111** | 0.529 | 0.522 | -0.380 | -0.607 |
| **D2 : ]111 - 145]** | 0.555 | 0.447 | -0.399 | -0.648 |
| **D3 : ]145 - 175]** | 0.595 | 0.495 | -0.323 | -0.635 |
| **D4 : ]175 - 210]** | 0.640 | 0.516 | -0.458 | -0.674 |
| **D5 : ]210 - 243]** | 0.620 | 0.483 | -0.505 | -0.708 |
| **D6 : ]243 - 280]** | 0.626 | 0.530 | -0.413 | -0.733 |
| **D7 : ]280 - 320]** | 0.612 | 0.519 | -0.321 | -0.673 |
| **D8 : ]320 - 378]** | 0.589 | 0.504 | -0.468 | -0.675 |
| **D9 : ]378 - 462]** | 0.652 | 0.589 | -0.429 | -0.755 |
| **D10 : > 461** | 0.625 | 0.601 | -0.371 | -0.757 |
| **(B) Deciles of animal to plant protein ratio** | **NRF 9.3^1^** | **MAR^1^** | **MER^1^** | **SED^1^** |
| **D1 : ≤ 1,68** | 0.605 | 0.591 | -0.376 | -0.705 |
| **D2 : ]1,68 - 2,08]** | 0.587 | 0.535 | -0.368 | -0.674 |
| **D3 : ]2,08 - 2,44]** | 0.659 | 0.593 | -0.376 | -0.695 |
| **D4 : ]2,44 - 2,76]** | 0.615 | 0.582 | -0.383 | -0.694 |
| **D5 : ]2,76 - 3,08]** | 0.610 | 0.547 | -0.372 | -0.691 |
| **D6 : ]3,08 - 3,41]** | 0.664 | 0.582 | -0.520 | -0.695 |
| **D7 : ]3,41 - 3,85]** | 0.551 | 0.519 | -0.322 | -0.755 |
| **D8 : ]3,85 - 4,44]** | 0.504 | 0.497 | -0.485 | -0.614 |
| **D9 : ]4,44 - 5,57]** | 0.661 | 0.545 | -0.500 | -0.684 |
| **D10 : > 5,57** | 0.523 | 0.473 | -0.345 | -0.595 |
| **(C) Deciles of “Alcoholic drinks” expenditure share** | **NRF 9.3^1^** | **MAR^1^** | **MER^1^** | **SED^1^** |
| **D1 : = 0** | 0.605 | 0.544 | -0.476 | -0.647 |
| **D2 : = 0** | 0.605 | 0.544 | -0.476 | -0.647 |
| **D3 : = 0** | 0.605 | 0.544 | -0.476 | -0.647 |
| **D4 : ]0 - 1,44]** | 0.658 | 0.572 | -0.453 | -0.672 |
| **D5 : ]1,44 - 3,19]** | 0.627 | 0.556 | -0.427 | -0.687 |
| **D6 : ]3,19 - 5,22]** | 0.569 | 0.500 | -0.311 | -0.694 |
| **D7 : ]5,22 - 8,39]** | 0.630 | 0.585 | -0.458 | -0.727 |
| **D8 : ]8,39 - 13,24]** | 0.612 | 0.574 | -0.374 | -0.709 |
| **D9 : ]13,24 - 21,21]** | 0.581 | 0.493 | -0.487 | -0.683 |
| **D10 : > 21,21** | 0.520 | 0.438 | -0.281 | -0.639 |
| **(D) Deciles of “Mixed dishes” expenditure share** | **NRF 9.3^1^** | **MAR^1^** | **MER^1^** | **SED^1^** |
| **D1 = 0.00** | 0.546 | 0.485 | -0.354 | -0.625 |
| **D2 : ]0.00 - 1.33]** | 0.531 | 0.511 | -0.252 | -0.702 |
| **D3 : ]1.33 - 2.57]** | 0.639 | 0.580 | -0.439 | -0.681 |
| **D4 : ]2.57 - 3.58]** | 0.589 | 0.541 | -0.382 | -0.739 |
| **D5 : ]3.58 - 4.99]** | 0.556 | 0.419 | -0.373 | -0.645 |
| **D6 : ]4.99 - 6.38]** | 0.662 | 0.553 | -0.485 | -0.732 |
| **D7 : ]6.38 - 8.49]** | 0.615 | 0.573 | -0.445 | -0.711 |
| **D8 : ]8.49 - 11.20]** | 0.618 | 0.607 | -0.414 | -0.671 |
| **D9 : ]11.20 - 15.89]** | 0.630 | 0.569 | -0.445 | -0.624 |
| **D10 > 15.89** | 0.583 | 0.527 | -0.408 | -0.585 |

^1^ All associations were significant at p<0.001

**Supplemental Figure S1:** Distribution of the revised Healthy Purchase Index (r-HPI) (A) in the external validation sample (n=2,188) of French households from the Kantar Worldpanel


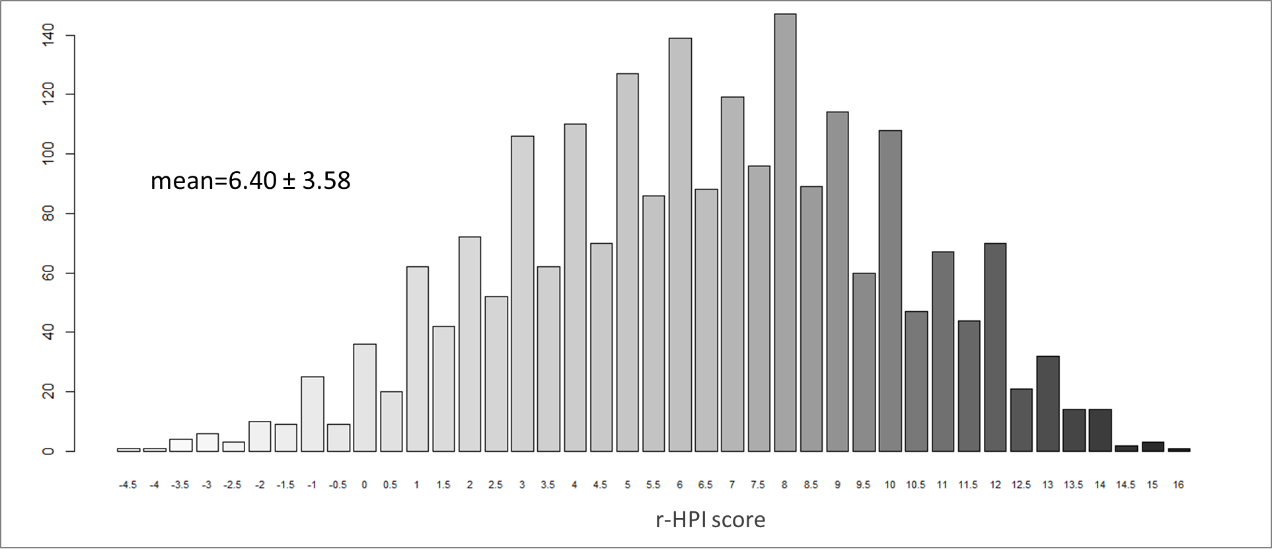


**Supplemental Figure S2**: Mean r-HPI by age (A), gender (B), and education level (C) of the respondent, income per consumption unit (D) and for households having a food basket of higher (MAR> median and MER and SED <median) vs. lower nutritional quality (E), in the external validation sample (n=2188) of French households from the Kantar Worldpanel. MAR: Mean Adequacy Ratio, MER: Mean Excess Ratio, r-HPI: revised Healthy Purchase Index, SED: Solid Energy Density


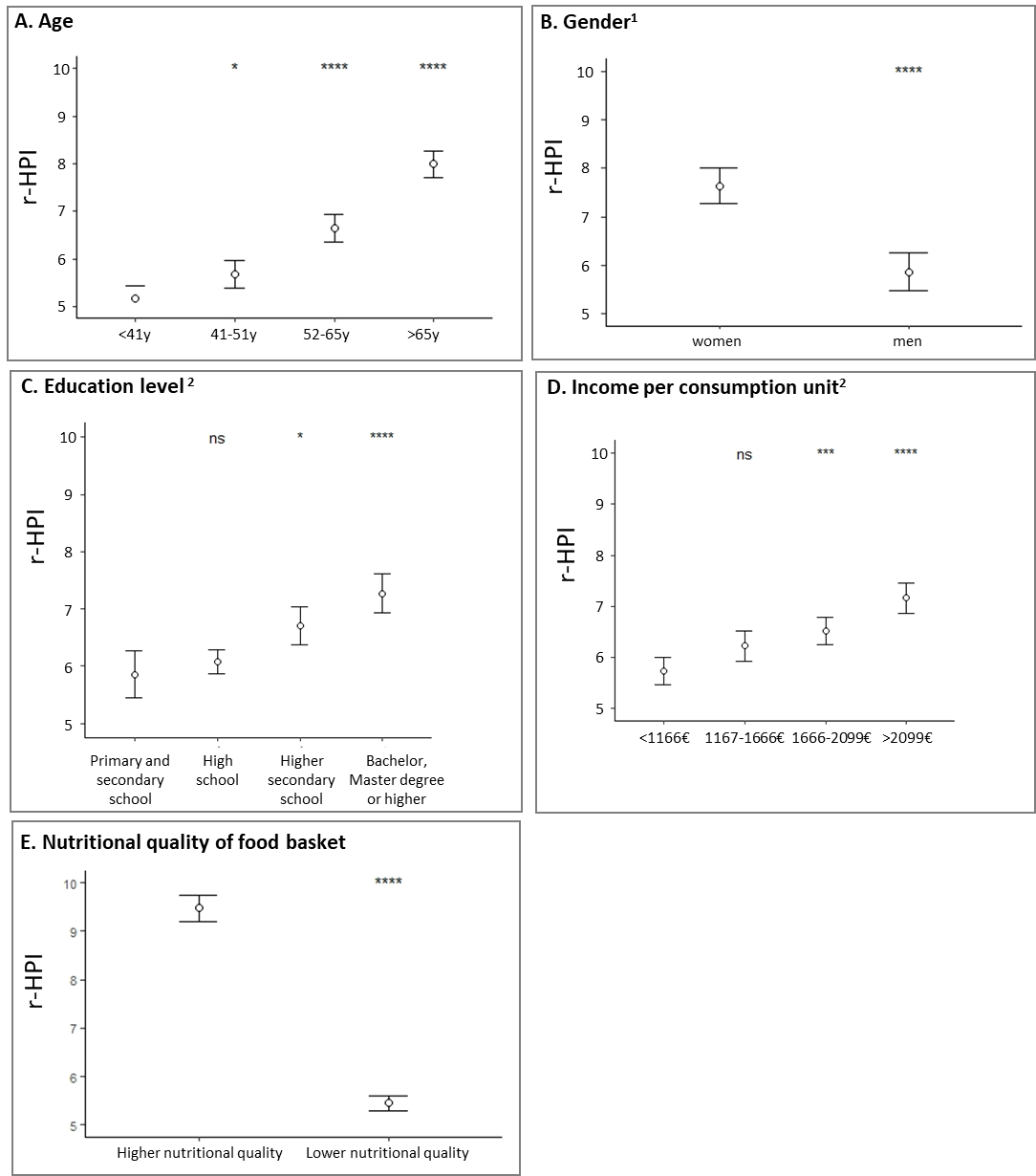


*p≤0.05, **p≤0.01, ***p≤0.001; ****p≤0.0001; ns: p>0.05 (Wilcoxon test or pairwise comparisons of contrasts according to the tested variable); ^1^ For gender, estimates and tests were performed on a subsample of single adult households (n=703); ^2^ Mean after adjustment for age
